# Pre-Exposure to Defibrotide Prevents Endothelial Cell Activation by Lipopolysaccharide: An Ingenuity Pathway Analysis

**DOI:** 10.3389/fimmu.2020.585519

**Published:** 2020-12-03

**Authors:** Nicoletta Orlando, Gabriele Babini, Patrizia Chiusolo, Caterina Giovanna Valentini, Valerio De Stefano, Luciana Teofili

**Affiliations:** ^1^Department of Image, Radiation therapy, Oncology and Hematology, Fondazione Policlinico Universitario A. Gemelli IRCCS, Rome, Italy; ^2^Department of Woman and Child Health and Public Health, Fondazione Policlinico Universitario A. Gemelli IRCCS, Rome, Italy; ^3^Department of Radiological and Hematological Sciences, Università Cattolica del Sacro Cuore, Rome, Italy

**Keywords:** endothelial cells, defibrotide, lipopolysaccharide, gene expression profile, ingenuity pathway analysis

## Abstract

Defibrotide (DFB) effects on different endothelial cell pathways have been investigated focusing on a limited number of genes or molecules. This study explored the modulation of the gene expression profile of steady-state or lipopolysaccharide (LPS)-activated endothelial cells, following the DFB exposure. Starting from differentially regulated gene expression datasets, we utilized the Ingenuity Pathway Analysis (IPA) to infer novel information about the activity of this drug. We found that effects elicited by LPS deeply differ depending on cells were exposed to DFB and LPS at the same time, or if the DFB priming occurs before the LPS exposure. Only in the second case, we observed a significant down-regulation of various pathways activated by LPS. In IPA, the pathways most affected by DFB were leukocyte migration and activation, vasculogenesis, and inflammatory response. Furthermore, the activity of DFB seemed to be associated with the modulation of six key genes, including matrix-metalloproteinases 2 and 9, thrombin receptor, sphingosine-kinase1, alpha subunit of collagen XVIII, and endothelial-protein C receptor. Overall, our findings support a role for DFB in a wide range of diseases associated with an exaggerated inflammatory response of endothelial cells.

## Introduction

Defibrotide (DFB) is an antithrombotic profibrinolytic drug characterized by anticoagulant activity and low hemorrhage risk (1). It consists of a polydisperse mixture of predominantly single-stranded polydeoxyribonucleotide sodium salts (2). DFB was formerly identified as a DNA fraction isolated from bovine lung, and currently, it is derived *via* controlled depolymerization of the porcine intestinal mucosal DNA (2). Since the first report of its clinical utilization in bone marrow transplant-associated veno-occlusive disease (VOD), DFB has come on the stage for its efficacy in such life-threatening condition (3, 4). Hepatic VOD, currently termed sinusoidal obstruction syndrome (SOS), is a potentially fatal complication occurring in hematological patients receiving conditioning regimens for hematopoietic stem cell transplantation (HSCT) or chemotherapy (4). DFB has been released in the United States for the treatment of VOD/SOS with renal or pulmonary dysfunction and in the European Union for severe VOD/SOS, respectively (5, 6). The exact mechanism by which DFB exerts its beneficial activity in this condition has been only partly deciphered, and probably it affects multiple endothelial pathways.

Endothelial colony-forming cells (ECFCs) are a rare cell population of circulating progenitors endowed with clonal potential, ability to give rise to mature endothelial cells, and promoting vascular formation *in vitro* and *in vivo* (7). The ECFC origin is still a matter of debate, whereas data on patients undergoing bone marrow transplantation suggested a bone-marrow origin (8, 9). The transcriptome profile of ECFCs resembles that of microvascular endothelial cells (10). Therefore, ECFCs have been used as an endothelial surrogate to investigate several hematological and extra-hematological conditions (11–15). Human derived-ECFCs have been proved to migrate to the liver and the intestine in a fetal sheep model of *in utero* transplantation, so contributing to the cytoarchitecture of these organs (16). Furthermore, in adult life, circulating endothelial progenitors repopulate sinusoidal endothelial cells during liver regeneration (17). Whereas VOD/SOS is generally reported in hematological patients, thrombotic microangiopathies can be observed outside of this setting (18). Since bacterial toxins, including lipopolysaccharide (LPS), may trigger thrombotic microangiopathies, we utilized ECFC cultures exposed to LPS as an experimental model of endothelial damage.

This study aimed to understand which endothelial pathways are mainly regulated by DFB, to gather further information for its potential therapeutic use.

## Material AND methods

### Study Design

The study was designed to explore the DFB-dependent *in vitro* effects in conditions mimicking *in vivo* administration for either preemptive or therapeutic DFB purposes. Before being exposed to LPS, endothelial cells were either or not preliminary incubated with DFB. DFB concentration used in the final experimental setting was preliminarily identified by evaluating the reduction of intercellular adhesion molecule-1 (ICAM-1) RNA expression after LPS exposure in the presence of increasing DFB concentrations (10, 50, 100 and 400 ng/ml, data not shown). We selected the concentration of 100 ng/ml since it was the lowest DFB concentration at which ICAM-1 RNA expression was maximally inhibited. This concentration reproduces the DFB plasma level achieved in the clinical setting after intravenous administration at recommended doses for VOD/SOS treatment (1, 2). The DFB used in the study was kindly provided by Jazz Pharmaceutical (Gentium S.P.A., Villa Guardia, CO, Italy).

### Cell Cultures and Conditions

ECFCs were obtained from cord blood units donated by the UNICATT Cord Blood Bank of Fondazione Policlinico Universitario A. Gemelli IRCCS (Rome, Italy) for transplant purposes. Units were collected from healthy full-term babies: units not suitable for transplantation for low volume or low cell content were used, according to the Institutional Ethics Committee approval (Prot. 0029871/16, July 20, 2016). ECFCs were obtained according to the original method of Ingram et al (7). Detailed methods for culture and endothelial cell characterization have been previously described (12). Briefly, ECFCs were grown in a growth basal medium (EBMTM-2, Lonza Group Ltd, Basel, Switzerland) with growth factor supplementation (EGMTM-2 Single Quots TM Supplements, Lonza Group Ltd) in 24-multiwell plates. The culture medium was replaced every 2 days. In order to minimize the effects induced by growth factors contained in the culture media (EGMTM-2 medium), all experiments were carried out in confluent cells (passages II to III) after 48 h from the last medium replace, in the presence of the basal medium (EBMTM-2 medium) containing 10% fetal bovine serum (Biowest, Nuaillé, France), 100 µg/ml streptomycin and 100 U/ml penicillin (both purchased from Merck Life Science S.r.l., Milan, Italy). LPS was purchased from Merck Life Science S.r.l and used at 100 ng/ml concentration in all experiments. Culture conditions were the following: **a**) **control cultures**: 6 h incubation in EBMTM-2; **b**) **DFB-cultures**: 6-h incubation in EBMTM-2 with DFB; **c**) **LPS-cultures**: 6-h incubation in EBMTM-2 with LPS; **d**) **LPS + DFB-cultures**: 6-h incubation in EBMTM-2 with LPS and DFB; **e) preDFB-LPS + DFB-cultures**: 2-h incubation in EBMTM-2 with DFB, followed by medium removal and then 6-h incubation in EBMTM-2 containing LPS and DFB. Cells were then washed in phosphate-buffered saline, directly lysed in the multiwell plates (Buffer RLT, Qiagen, Germantown, MD, USA), and used for RNA extraction. A minimum of six overall experiments were carried out for each condition and in each set of experiments ECFCs derived from one single cord blood unit were utilized.

### Gene Expression Profiling and Analysis

Total RNA was extracted using the RNaesy MiniKit (Qiagen, Germantown, MD, USA), according to manufacturer instructions. QIAxpert Spectrophotometer (Qiagen) was used to assess RNA integrity and concentration. Reverse transcription was performed with 0.5 µg of total RNA using the RT^2^ First Strand Kit (Qiagen). Real-time PCR was carried out by RT^2^ SYBR Green ROX qPCR Mastermix (Qiagen) using the Stratagene Mx3000P Real-Time PCR detection system (Agilent Technologies, Santa Clara, CA, USA). Gene expression profile was assessed by the Human Endothelial Cell Biology RT^2^ Profiler PCR Array (PAH 015Z, Qiagen), including primers for 84 tests and five housekeeping genes (**Supplementary Table 1**). Controls were included to assess reverse transcription efficiency PCR reaction performance and genomic DNA contamination. Relative changes in gene expression were calculated accordingly to the 2^−ΔΔCT^ method using the cycle threshold (CT) obtained at real-time PCR. Data analysis was performed using the web-based PCR array data analysis software, available at https://dataanalysis2.qiagen.com/pcr.

### Statistical and Bioinformatics Analysis

Mean expression values of the genes for the five treatment conditions were considered as input values for a heatmap plot by the online tool Heatmapper (Clustering method: Complete Linkage, Distance Measurement Method: Spearman Rank Correlation) (19). Statistical comparison was performed using the Student’s t-test of the replicate 2^(-Delta CT). Given the inter-individual variability between the ECFCs, p-values less than 0.1 were considered statistically significant for the pathway analysis. In order to highlight the underlying mechanisms regulating the observed changes in gene expression profiles, *in silico* analyses with Ingenuity Pathway Analysis (IPA, Qiagen) were performed. Data relative to genes with an average threshold cycle either not determined or greater than the defined cut-off (default 35), in investigated samples and controls were excluded from the analysis (**Table 1 Supplementary**). Three types of analysis were performed in IPA: 1) **downstream effect analysis** canonical including pathways analysis (to compare well-established signaling pathways) and bio-functions’ analysis (to compare activation or inhibition of critical biological processes or functions), 2) **upstream regulators’ analysis**, *i.e.* likely regulators that are connected to dataset genes through a set of direct and indirect relationships 3) **interaction and causal network analysis**, *i.e.* a generalized analysis of additional regulators that connect upstream regulators to genes significantly modulated in experimental datasets, including intermediate regulators (20). In order not to limit the study only to the five treatment conditions of our *in vitro* model, IPA filter settings were not restricted to endothelial cells only. Results were expressed as Z-score (*i.e.* the match of observed and predicted up/down regulated patterns) (20).

All relevant data are contained within the article. The original contributions presented in the study are included in the supplementary material and further inquiries can be directed to the corresponding author.

## Results

**Figure 1:**
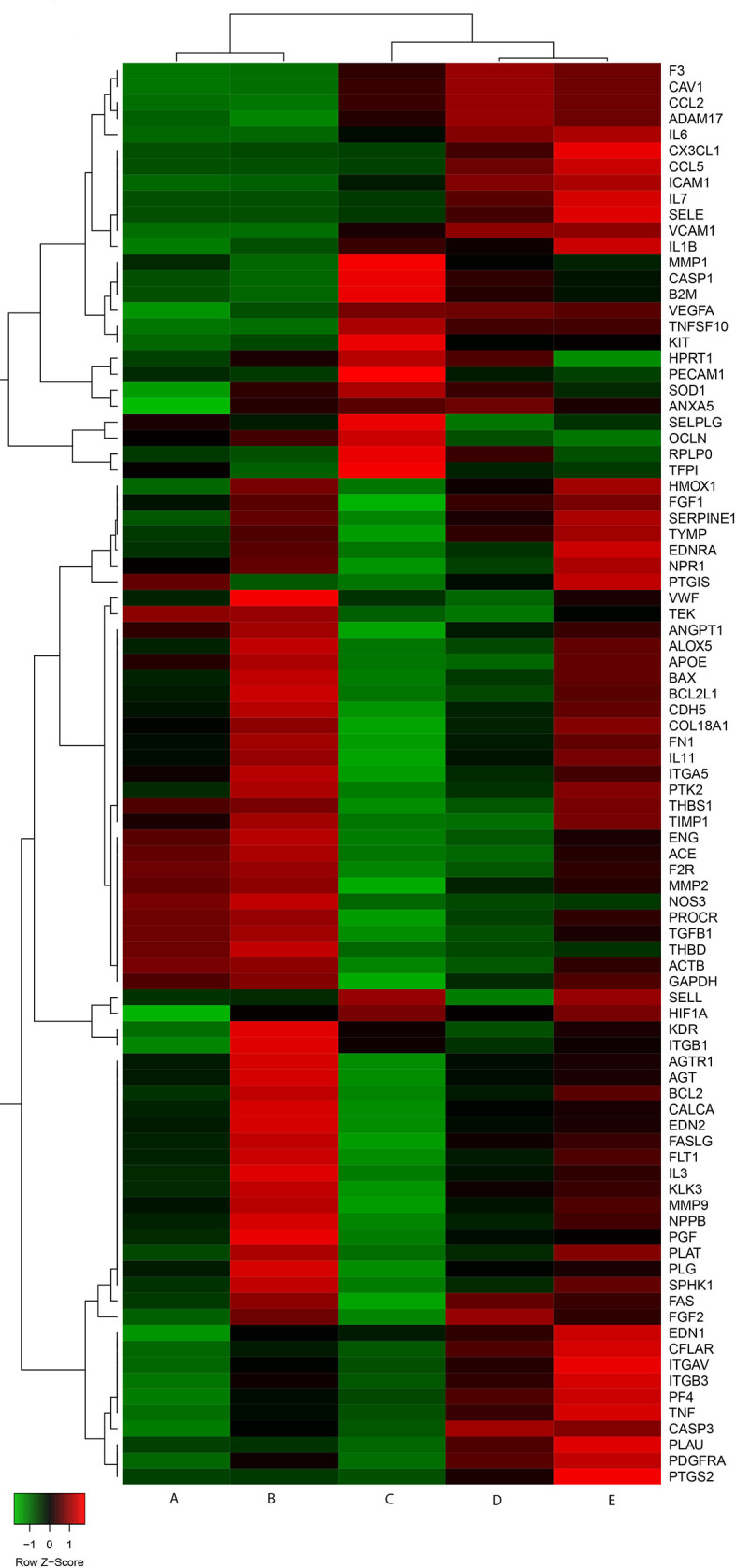
Gene expression heatmap of cells in various experimental conditions. Control cells **(A)** and cells exposed to defibrotide **(B)** are clustered together. Similarly behave cells pre-exposed to defibrotide, and subsequently to lipopolysaccharide **(C)**, and cells exposed to lipopolysaccharide **(D)** or lipopolysaccharide and defibrotide **(E)**. Nevertheless, the cluster shows a further branching between cells exposed to lipopolysaccharide (with or without defibrotide, corresponding to **(E**, **D)**, respectively) and those subjected to defibrotide pre-incubation **(C)**.

We carried out a preliminary exploratory heatmap analysis to evaluate gene expression profile clustering in different experimental conditions (**Figure 1**). Two principal branches were identified, with the left branch including control cultures and endothelial cells exposed to DFB and the right one cells exposed to LPS, to LPS and DFB together, or pre-exposed to DFB and then to LPS and DFB. Noteworthy, LPS-cultures and LPS + DFB-cultures clustered together, whereas the preliminary DFB exposure induced an expression profile intermediate between controls and cells cultured in presence of LPS + DFB (**Figure 1**).

### Gene Expression and Ingenuity Pathway Analysis of Steady-State Cells Exposed to Defibrotide

**Figure 2:**
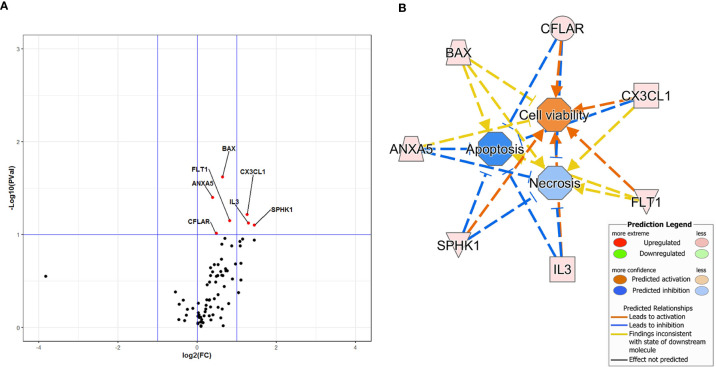
Volcano Plot analysis of 78 genes expressed in cells exposed to defibrotide in comparison with controls **(A)**. The plot shows the log2-Fold Change (FC) on the *x*-axis, and the negative log10 of *p*-values on the *y*-axis. Color-coding is based on the fold change. Thick vertical lines highlight fold changes of −2 and +2, while a thick horizontal line represents a p-value of 0.1. Red dots: fold changes ≥1 and *p*-values ≤0.1; green dots: fold changes <1 and *p*-values ≤0.1; black dots: not-significant *p*-values. **(B)** Downstream effects predicted through the Ingenuity Pathway Analysis of the gene dataset of defibrotide-exposed cells. An enhancement of cell viability and inhibition of apoptosis and necrosis are expected because of defibrotide exposure.

The effect of DFB on gene expression profile of steady-state cells is illustrated in the Volcano plot of **Figure 2A**. Seven out of 78 investigated genes were significantly upregulated, with a fold change ranging from 1.31 to 2.71 (**Figure 2A**). Downstream effect analysis suggested that these genes are mainly connected to cell viability, necrosis, and apoptosis (activation Z-score 1.18 for cell viability, −1.20 for apoptosis and −0.52 for necrosis) with a predicted increase of cell viability and decrease of necrosis and apoptosis, as indicated by IPA function analysis (**Figure 2B**).

### Gene Expression Analysis of Cells Exposed to Lipopolysaccharide and Defibrotide

**Figure 3:**
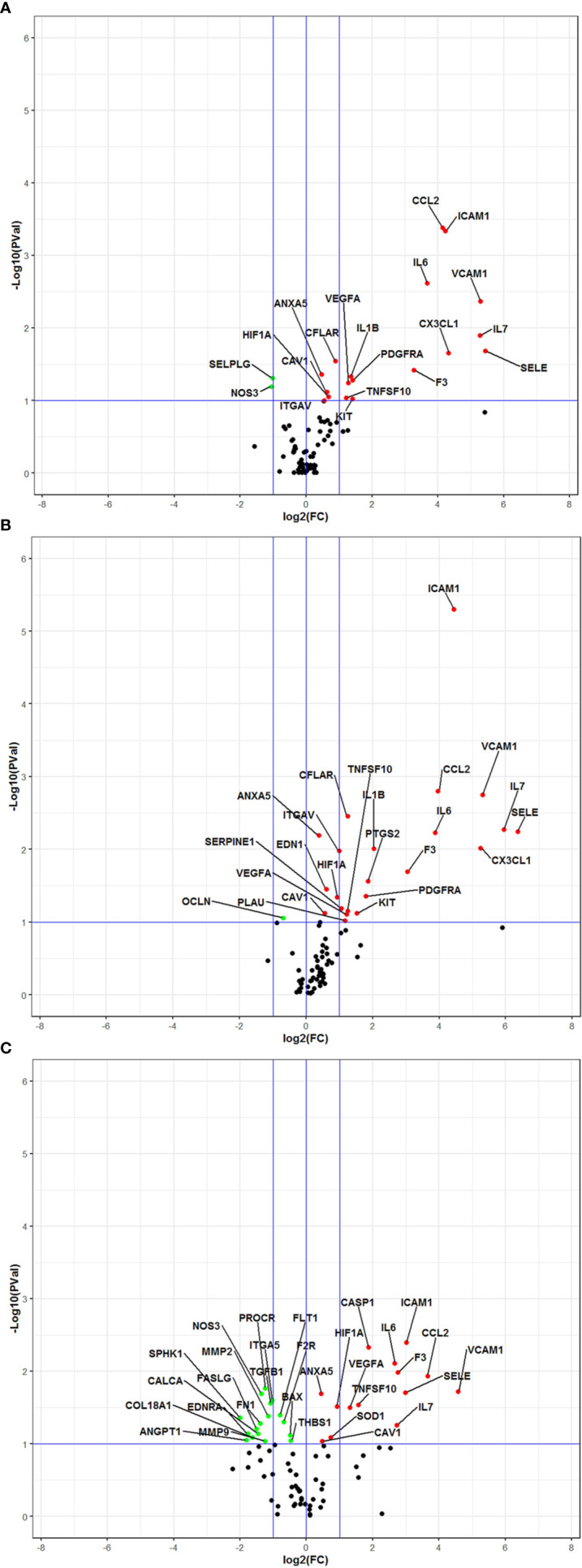
Volcano Plot analysis of 78 genes expressed in various culture conditions. All data are intended in comparison with control cultures **(A)** Cells exposed to lipopolysaccharide. **(B)** Cells exposed to lipopolysaccharide and defibrotide. **(C)** Cells pre-exposed to defibrotide, and then to lipopolysaccharide. The plots show the log2-Fold Change (FC) on the 𝑥-axis and the negative log10 of *p*-values on the *y*-axis. Color coding is based on the fold change. Thick vertical blue lines indicate fold changes of −2 and +2; thick horizontal blue lines indicate the p-value of 0.1. Red dots: fold changes ≥1 and *p*-values ≤0.1; green dots: fold changes <1 and *p*-values ≤0.1; black dots: not-significant *p*-values.

The complete list of investigated genes and their expression in the different experimental settings in comparison with controls is provided in **Supplementary Table 2**. As expected, cells exposed to LPS displayed significant changes of expression in a relevant number of genes, including CCL2, CX3CL1, F3, IL6, IL7, SELE, ICAM1, VCAM, PDGFRA, KIT, and VEGFA, with a fold change ranging from 1.38 to 43.24 (**Figure 3A** and **Supplementary Table 2**). Significant downregulation of NOS3 and SELPG expressions were also observed, with a fold change of −2.07 and −2.02 respectively (**Figure 3A** and **Supplementary Table 2**). The gene expression profile of cells exposed to LPS + DFB was very similar to that of cells exposed to the LPS only, with 23 genes significantly deregulated: 22 of them were upregulated (fold change range 1.30–81.93) while one gene (OCLN) was downregulated with a fold change of −1.61 (**Figure 3B** and **Supplementary Table 2**). The trend of expression of genes in cells exposed to LPS and in those cultured with LPS + DFB was often consensual, whereas the intensity of gene modulation and statistical significance of the observed effect were slightly different in the two conditions. Conversely, the DFB pre-exposure deeply modified the pattern of gene expression. When endothelial cells were preemptively incubated with DFB, LPS exposure produced a significant modulation of 31 genes: 14 of them were upregulated (fold change ranging from 1.36 to 23.78), whereas additional 17 genes were downregulated (fold change ranging from −1.39 to −3.98) (**Figure 3C** and **Supplementary Table 2**).

### Ingenuity Pathway Analysis of Cells Exposed to Lipopolysaccharide and Defibrotide

**Figure 4:**
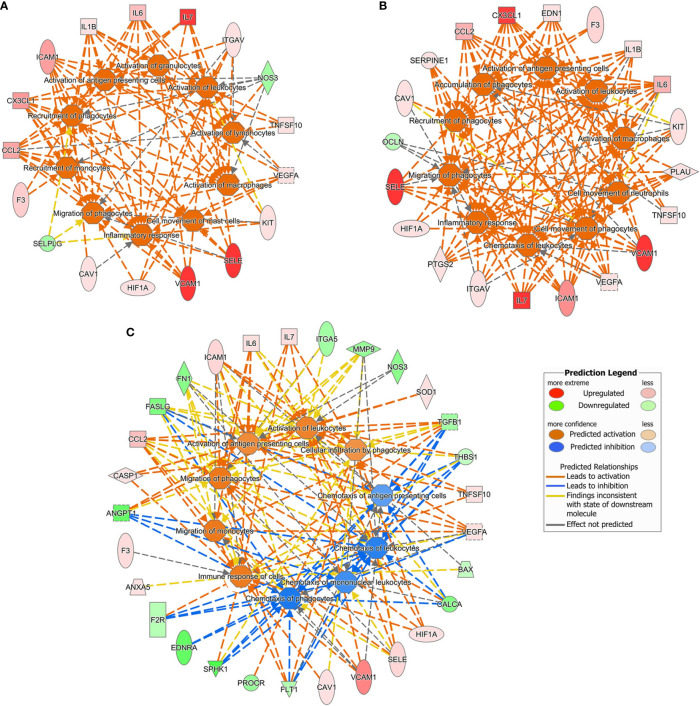
Ten top-most predicted activated or inhibited downstream bio functions according to the Ingenuity Pathway Analysis. Gene datasets obtained in different culture conditions were uploaded. **(A)** Cells exposed to lipopolysaccharide. **(B)** Cells exposed to lipopolysaccharide and defibrotide. **(C)** Cells pre-exposed to defibrotide, and then to lipopolysaccharide.

Since the IPA setting was not restricted to endothelial cells, the analysis of gene expression changes in our datasets led to predict also bio-functions in other types of cells and tissues. The ten top-most most affected pathways (upstream regulators, diseases and functions, and canonical pathways) in various experimental settings are illustrated in **Figure 4** and **Table 1**. The gene expression changes due to LPS either alone or combined with DFB, predicted the activation of the immune response, migration of monocytes and phagocytes, activation of leukocytes and antigen-presenting cells, (activation Z-score range: 2.19–3.14), while no pathway was downregulated (**Figures 4A, B****)**. The DFB pre-exposure significantly damped the activation of the above-mentioned pathways, likewise indicating a parallel inhibition of blood cell chemotaxis (**Figure 4C**). Whereas many of the upstream regulators (including F2, LPS, TNF, and IFN*γ*), appeared to be common drivers of cell response to LPS and LPS + DFB, their role in DFB-pre-exposed cells was much less evident, with very low Z-score values (**Table 1**). Similarly, several bio-functions that were clearly activated in cells exposed to LPS or LPS + DFB were only weakly activated or at all inhibited (with negative Z-score values), when cells were preemptively exposed to DFB (**Table 1**). In particular, the bio-functions on which DFB pre-exposure exerted the strongest inhibition were “inflammatory response”, “cell movement”, and “chemotaxis”. Similar results were observed regarding canonical pathways. Six pathways were significantly up-regulated by LPS: High Mobility Group-B1 (HMGB1) signaling; Hepatic Fibrosis Signaling Pathway, Neuroinflammation Signaling Pathway, Triggering Receptor Expressed on Myeloid cells 1 (TREM1) signaling; IL-8 Signaling and Apelin Endothelial Signaling Pathway (**Table 1**). While the contemporary DFB exposure slightly modified the activation of these pathways, almost all showed lower Z-score values if cells were pre-incubated with DFB (**Table 1**).

**Table 1:** Ingenuity pathway analysis of gene expression datasets gathered in different experimental settings.

	LPS	LPS+DFB	DFB pre-exposed
**Upstream Regulators**			
lipopolysaccharide	3.751	4.104	0.574
F2	3.682	3.942	1.047
IL-1B	3.506	3.638	1.823
IKBKB	3.372	3.655	1.080
TNF	3.358	4.031	1.534
tetradecanoylphorbol acetate	3.303	3.242	1.163
D-glucose	3.272	3.844	0.349
IFNG	3.268	3.783	1.859
poly rI:rC-RNA	3.188	3.265	1.039
CD40	3.098	3.400	2.194
**Bio Functions**			
Migration of cells	3.305	3.877	0.628
Leukocyte migration	3.191	3.523	0.866
Activation of leukocytes	3.135	3.437	1.373
Vasculogenesis	3.064	3.791	0.542
Inflammatory response	3.051	3.752	−0.178
Chemotaxis	3.006	3.834	−0.862
Adhesion of immune cells	2.960	3.111	2.069
Occlusion of artery	2.952	2.939	1.004
Atherosclerosis	2.952	2.939	0.775
Cell movement of tumor cell lines	2.925	3.516	−0.671
**Canonical pathway**			
HMGB1 Signaling	2.449	2.449	1.633
Hepatic Fibrosis Signaling Pathway	2.646	3.000	0.000
Neuroinflammation Signaling Pathway	2.646	2.121	0.707
TREM1 Signaling	2.000	2.000	1.342
IL-8 Signaling	2.000	2.236	0.000
Apelin Endothelial Signaling Pathway	1.342	2.000	0.816

For each setting is reported the Z-score relative to the 10 top-most predicted activated or inhibited upstream regulators, bio functions, and canonical pathways.

DFB, defibrotide; LPS, lipopolysaccharide; F2, coagulation Factor II, Thrombin; HMGB1, high mobility group protein B1; IFNG, interferon-gamma; IKBKB, inhibitor of nuclear factor-kappa B kinase subunit beta; IL, Interleukin; TNF, tumor necrosis factor.

### Network Analysis

**Figure 5:**
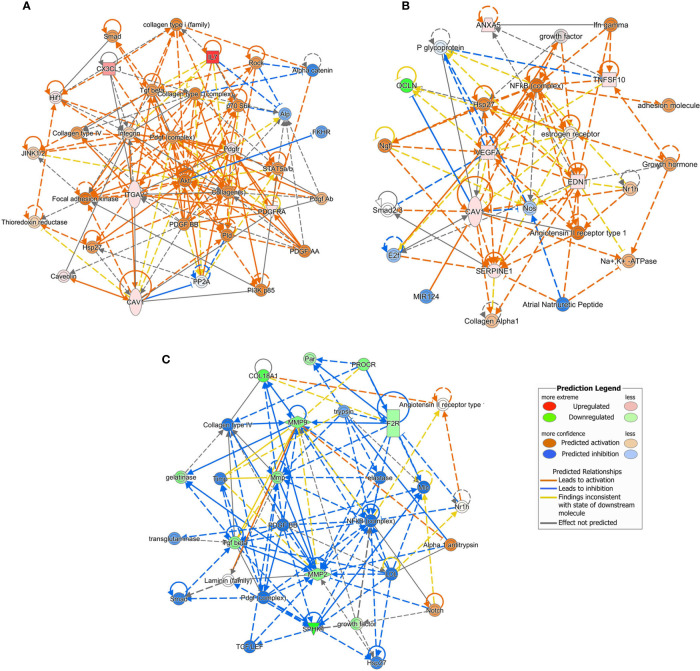
Causal network analysis obtained at Ingenuity Pathway Analysis of different gene datasets. The highest-score network is displayed for each culture condition. **(A)** Cells exposed to lipopolysaccharide. **(B)** Cells exposed to lipopolysaccharide and defibrotide. **(C)** Cells pre-exposed to defibrotide, and then to lipopolysaccharide. The relationship among molecules is represented by lines (solid lines for direct association and dotted lines for indirect association).

For each experimental condition, we generated enriched interaction networks based on known relationships in the Qiagen Knowledge Base and ordered by decreasing IPA score. We identified six networks for LPS exposed cells, six networks for cells exposed to LPS with DFB, and eight networks for cells pre-exposed to DFB and then to LPS with DFB. The highest score network (score 11) in the first condition (LPS) was centered on five genes (CAV1, CX3CL1, IL7, ITGAV, PDGFRA) (**Figure 5A**). The highest score network (score 16) in cells incubated with LPS + DFB was centered on seven genes (Serpine1, CAV1, EDN1, ANXA5, OCLN, VEGFA, TNGSF10) (**Figure 5B**). The highest score network (score 12) in DFB pre-exposed cells was centered on six genes (COL18A1, F2R, MMP2, MMP9, PROCR, and SPHK1) (**Figure 5C**).

## Discussion

This study explored gene expression changes occurring in steady state or LPS-activated endothelial cells exposed to DFB. Starting from these datasets, we utilized IPA functions to infer novel information about the activity of this drug. We found that effects elicited by LPS deeply differ depending whether cells are incubated with DFB and LPS at the same time, or if the DFB priming occurs before the LPS exposure. In fact, only in the second condition, we could observe a significant down-regulation of various pathways activated by LPS. Moreover, our results suggest that the anti-inflammatory activity of DFB exceeds its antithrombotic effect. Since IPA blankets a wide range of pathways and bio-functions, we could therefore demonstrate that defibrotide exerts a greater effect on the inflammatory response than on pathways related to blood coagulation.

From a functional standpoint, the progeny of ECFCs that we used in our study is comparable to microvascular endothelial cells (10). These cells share a common origin with hematopoietic precursors and circulate in peripheral blood to reach the injured endothelium (7, 9). In this setting LPS exposure induces the gene overexpression of inflammatory cytokines, tissue factor, chemokines, and adhesion molecules, associated with the downregulation of NOS3, a strong endogenous inhibitor of leukocyte adhesion (21). The exposure to DFB results in the overexpression of genes with known vascular protective effects, promoting endothelial cell integrity, survival and proliferation, such as ANXA5 (22), CFLAR (23), IL3 (24), and FLT1 (25). Nevertheless, we observed also a concurrent overexpression of additional genes generally related to the inflammatory response, such as SPHK1, CX3CL1, and BAX (26, 27). In our experimental setting, DFB seemed unable to offset the LPS effects once LPS-induced pathways were activated. In fact, cells either exposed to LPS, alone or combined to DFB, displayed similar gene expression profiles, upstream regulators and downstream effects. Conversely, the preliminary incubation with DFB significantly damped the effects of LPS. It is conceivable that DFB might compete with LPS for some pathways. The significant overexpression of SPHK1 and CX3CL1 observed in cells exposed to DFB alone seems to support this hypothesis (26, 27). Previous study demonstrated that DFB is internalized by endothelial cells but does not enter nuclei: the DFB attachment on the cytoplasmic cell membrane is sufficient to elicit its effect (28). Therefore, it is conceivable that the preliminary exposure of cells to DFB may prevent further activation of specific cell pathways by LPS, impeding at membrane level the LPS interaction with definite pattern-recognition receptors, such as Toll-like receptors (29).

At variance with many previous reports focusing specific molecules, this study explored a comprehensive panel of genes, involved in many different biological functions of endothelial cells. In addition, IPA compared gene expression changes among different datasets and extrapolated which molecular pathways and cell bio-functions were predominantly modulated in each experimental condition. Previous studies showed that DFB promotes the expression of thrombomodulin (30), reduces the production of tissue factor, and plasminogen activator inhibitor-1, and likewise increases the expression tissue type-plasminogen activator (31, 32). In addition, it has been demonstrated that DFB influences adhesive properties of endothelial cells through reducing selectin and ICAM-1 expression and modulates the extracellular matrix composition by increasing heparanase production (33–35). Furthermore, DFB prevents endothelial cell from apoptosis triggered by a wide range of stimuli, attenuates the inflammatory response, and reduces immune alloreactivity (32–34). Results that we achieved by IPA suggested that the bio-functions most significantly inhibited by DFB in LPS-activated cells were “inflammatory response”, “cell recruitment” and “adhesion”. Regarding blood coagulation, IPA includes three main bio functions, named “thrombus” “coagulation of blood” and “hemostasis”, respectively. Although IPA did not list them among the most affected by DFB, in all cases the Z-scores calculated in datasets of DFB-pretreated cells were always lower than in cells exposed to LPS, either alone or in combination with DFB. This finding suggests that these pathways were less activated in DFB-pretreated endothelial cells. In our experimental setting, DFB reduced the expression of tissue factor, increased the coagulation inhibitor TFPI and reduced the fibrinolysis inhibitor SERPINE-1. Among these effects, however, only tissue factor downregulation was statistically significant (**Supplementary Table 2**). It should be mentioned that previous data were gathered on different types of human endothelial cells, including macrovascular endothelial cells isolated from umbilical veins, dermal microvascular endothelial cells, or endothelial cell lines. Moreover, different types of compounds besides lipopolysaccharide (31) were used as activating stimuli, including thalidomide (32), fludarabine (33), cyclosporine (35), tacrolimus (35), or even soluble factors released by hematopoietic transplanted cells (34), Indeed, different experimental conditions, in addition to the diverse analysis approach, might account for the dissimilarities between our and previous observations.

IPA allowed us to identify which genes were the putative master regulators of networks principally affected by DFB. In particular, IPA rated as the most reliably responsible gene network for the pre-emptive LPS-inhibitory activity of DFB that including F2R, MMP2, MMP9, PROCR, SPHK1, and COL18A1. These genes are interconnected by multiple and complex relationships. All of them are implicated in the maintenance of cell integrity during the inflammatory response (36, 37). Matrix-metalloproteinase (MMP) exerts an extensive remodeling activity of the extracellular matrix, and process chemokines, cytokines, and cell surface receptors (38). MMP2 is a known activator of F2R, and prompts F2R interaction with PROCR on cell surface (39, 40). MMP role has been investigated in several acute and chronic inflammatory diseases, leading to the conclusion that MMP dysfunctions in specified organs may play a pathogenic role (38). Interestingly, the liver-selective MMP9 inhibition in a knockdown MMP9 rat model prevents proteolytic cleavage of hepatic VEGF, enhances recruitment and engraftment of bone marrow endothelial progenitors and accelerates liver regeneration after hepatectomy (41). It has been shown that monocrotaline, a pyrrolizidine alkaloid that induces SOS/VOD in both humans and experimental animals, increases in rat sinusoidal endothelial cells the expression of MMP9 and MMP2 (42). Notably, MMP9 and MMP2 inhibition by doxycycline prevents the development of SOS/VOD (42).

Altogether, our data provide the rationale for a stronger efficacy of DFB when preemptively used. In this regard, our observations agree with previous studies reporting a better outcome of VOD/SOS in HSCT patients early receiving DFB (43, 44). Similarly, the efficacy of DFB prophylaxis for VOD/SOS has been documented (45). Moreover, our analysis predicts that DFB, besides hindering the LPS effects, inhibits additional upstream regulators of the inflammatory response, such as INF-*γ*, TNF, IL1B, and CD40. These data have a relevant clinical impact and support a possible role for DFB in a wide range of diseases sustained by or associated with preeminent endothelial damage, such as severe infections, thrombotic microangiopathies, and allograft immune reactions (37). In fact, the ability of DFB to prevent the endothelial pro-inflammatory response to exogenous injury can limit endothelial cell activation, restraining the generation of a pro-inflammatory environment which further amplifies the endothelial cell damage.

In conclusion, using the different IPA approaches and functions, we expanded current knowledge about mechanisms responsible for the therapeutic effects of DFB. The findings gathered in this study constitute the basis for future trial exploring the DFB efficacy in several diseases whose pathogenesis relies on the endothelial dysfunction as pathogenic mechanism.

## Data Availability Statement

The original contributions presented in the study are included in the article/**Supplementary Material**, further inquiries can be directed to the corresponding author.

## Author Contributions

NO performed the experiments, collected, and analyzed the data, and wrote and approved the final manuscript. GB analyzed the data, and reviewed and approved the final manuscript. PC analyzed the data, and reviewed and approved the final manuscript. CGV collected the data and approved the final manuscript. VS critically reviewed and approved the final manuscript. LT designed the study, collected and analyzed the data, and wrote and approved the final manuscript. All authors contributed to the article and approved the submitted version.

## Funding

The study was funded by Jazz Pharmaceutical (grant number IST-16-10224). The funder was not involved in the study design, collection, analysis, interpretation of data, the writing of this article or the decision to submit it for publication.

## Conflict of Interest

The authors declare that the research was conducted in the absence of any commercial or financial relationships that could be construed as a potential conflict of interest.

## Abbreviations

ANXA5, Annexin A5; BAX, BCL2-associated X protein; CAV1, Caveolin 1; CCL2, Chemokine (C-C motif) ligand 2; CFLAR, CASP8 and FADD-like apoptosis regulator; COL18A1, Collagen, type XVIII, alpha 1; CX3CL1, Chemokine (C-X3-C motif) ligand 1; DFB, Defibrotide; ECFCs, Endothelial colony-forming cells; EDN1, Endothelin 1; F2, Coagulation Factor II (Thrombin); F2R, Coagulation factor II (thrombin) receptor; F3, Coagulation factor III (thromboplastin, tissue factor); FLT1, Fms-related tyrosine kinase 1; ICAM-1, Intercellular adhesion molecule-1; IFN*γ*, Interferon gamma; IL, Interleukin; ITGAV, Integrin, alpha V; LPS, Lipopolysaccharides; MMP, Matrix-metalloproteinase; NOS3, Nitric oxide synthase 3; OCLN, Occludin; KIT, V-kit Hardy–Zuckerman 4 feline sarcoma viral oncogene homolog; PDGFRA, Platelet-derived growth factor receptor alpha; PROCR, Endothelial-protein C receptor; SELE, Selectin Endothelial; SELPG, Selectin platelet ligand; SERPINE1, Serpin peptidase inhibitor-1; SOS, Sinusoidal obstruction syndrome; SPHK1, Sphingosine kinase 1; TFPI, Tissue factor pathway inhibitor; TNF, Tumor necrosis factor; TNGSF10, Tumor necrosis factor (ligand) superfamily, member 10; VCAM, Vascular cell adhesion molecule 1; VEGFA, Vascular endothelial growth factor A; VOD, Veno-occlusive disease.
