# Supplementary material for: Pre-Exposure to Defibrotide Prevents Endothelial Cell Activation by Lipopolysaccharide: An Ingenuity Pathway Analysis
Source: Front Immunol. 2020 Dec 3;11:585519. doi: 10.3389/fimmu.2020.585519 (PMC7744778; doi:10.3389/fimmu.2020.585519)
Supplement: Supplementary file 1 [file Table_1.docx]

**Supplementary Table 1.** Genes included in Human Endothelial Cell Biology RT^2^ Profiler PCR Array.

| **Symbol** | **Description** |
| --- | --- |
| ACE | Angiotensin I converting enzyme (peptidyl-dipeptidase A) 1 |
| ADAM17 | ADAM metallopeptidase domain 17 |
| AGT | Angiotensinogen (serpin peptidase inhibitor, clade A, member 8) |
| AGTR1 | Angiotensin II receptor, type 1 |
| ALOX5 | Arachidonate 5-lipoxygenase |
| ANGPT1 | Angiopoietin 1 |
| ANXA5 | Annexin A5 |
| APOE | Apolipoprotein E |
| BAX | BCL2-associated X protein |
| BCL2 | B-cell CLL/lymphoma 2 |
| BCL2L1 | BCL2-like 1 |
| CALCA | Calcitonin-related polypeptide alpha |
| CASP1 | Caspase 1, apoptosis-related cysteine peptidase (interleukin 1, beta, convertase) |
| CASP3 | Caspase 3, apoptosis-related cysteine peptidase |
| CAV1 | Caveolin 1, caveolae protein, 22kDa |
| CCL2 | Chemokine (C-C motif) ligand 2 |
| CCL5 | Chemokine (C-C motif) ligand 5 |
| CDH5 | Cadherin 5, type 2 (vascular endothelium) |
| CFLAR | CASP8 and FADD-like apoptosis regulator |
| COL18A1 | Collagen, type XVIII, alpha 1 |
| CX3CL1 | Chemokine (C-X3-C motif) ligand 1 |
| EDN1 | Endothelin 1 |
| EDN2 | Endothelin 2 |
| EDNRA | Endothelin receptor type A |
| ENG | Endoglin |
| F2R | Coagulation factor II (thrombin) receptor, PAR-1 |
| F3 | Coagulation factor III (thromboplastin, tissue factor) |
| FAS | Fas (TNF receptor superfamily, member 6) |
| FASLG | Fas ligand (TNF superfamily, member 6) |
| FGF1 | Fibroblast growth factor 1 (acidic) |
| FGF2 | Fibroblast growth factor 2 (basic) |
| FLT1 | Fms-related tyrosine kinase 1 (vascular endothelial growth factor/vascular permeability factor receptor) |
| FN1 | Fibronectin 1 |
| HIF1A | Hypoxia inducible factor 1, alpha subunit (basic helix-loop-helix transcription factor) |
| HMOX1 | Heme oxygenase (decycling) 1 |
| ICAM1 | Intercellular adhesion molecule 1 |
| IL11 | Interleukin 11 |
| IL1B | Interleukin 1, beta |
| IL3 | Interleukin 3 (colony-stimulating factor, multiple) |
| IL6 | Interleukin 6 (interferon, beta 2) |
| IL7 | Interleukin 7 |
| ITGA5 | Integrin, alpha 5 (fibronectin receptor, alpha polypeptide) |
| ITGAV | Integrin, alpha V (vitronectin receptor, alpha polypeptide, antigen CD51) |
| ITGB1 | Integrin, beta 1 (fibronectin receptor, beta polypeptide, antigen CD29 includes MDF2, MSK12) |
| ITGB3 | Integrin, beta 3 (platelet glycoprotein IIIa, antigen CD61) |
| KDR | Kinase insert domain receptor (a type III receptor tyrosine kinase) |
| KIT | V-kit Hardy-Zuckerman 4 feline sarcoma viral oncogene homolog |
| KLK3 | Kallikrein-related peptidase 3 |
| MMP1 | Matrix metallopeptidase 1 (interstitial collagenase) |
| MMP2 | Matrix metallopeptidase 2 (gelatinase A, 72kDa gelatinase, 72kDa type IV collagenase) |
| MMP9 | Matrix metallopeptidase 9 (gelatinase B, 92kDa gelatinase, 92kDa type IV collagenase) |
| NOS3 | Nitric oxide synthase 3 (endothelial cell) |
| NPPB | Natriuretic peptide B |
| NPR1 | Natriuretic peptide receptor A/guanylate cyclase A (atrionatriuretic peptide receptor A) |
| OCLN | Occludin |
| PDGFRA | Platelet-derived growth factor receptor, alpha polypeptide |
| PECAM1 | Platelet/endothelial cell adhesion molecule |
| PF4 | Platelet factor 4 |
| PGF | Placental growth factor |
| PLAT | Plasminogen activator, tissue |
| PLAU | Plasminogen activator, urokinase |
| PLG | Plasminogen |
| PROCR | Protein C receptor, endothelial |
| PTGIS | Prostaglandin I2 (prostacyclin) synthase |
| PTGS2 | Prostaglandin-endoperoxide synthase 2 (prostaglandin G/H synthase and cyclooxygenase) |
| PTK2 | PTK2 protein tyrosine kinase 2 |
| SELE | Selectin E |
| SELL | Selectin L |
| SELPLG | Selectin P ligand |
| SERPINE1 | Serpin peptidase inhibitor, clade E (nexin, plasminogen activator inhibitor type 1), member 1 |
| SOD1 | Superoxide dismutase 1, soluble |
| SPHK1 | Sphingosine kinase 1 |
| TEK | TEK tyrosine kinase, endothelial |
| TFPI | Tissue factor pathway inhibitor (lipoprotein-associated coagulation inhibitor) |
| TGFB1 | Transforming growth factor, beta 1 |
| THBD | Thrombomodulin |
| THBS1 | Thrombospondin 1 |
| TIMP1 | TIMP metallopeptidase inhibitor 1 |
| TNF | Tumor necrosis factor |
| TNFSF10 | Tumor necrosis factor (ligand) superfamily, member 10 |
| TYMP | Thymidine phosphorylase |
| VCAM1 | Vascular cell adhesion molecule 1 |
| VEGFA | Vascular endothelial growth factor A |
| VWF | Von Willebrand factor |
| ACTB | Actin, beta |
| B2M | Beta-2-microglobulin |
| GAPDH | Glyceraldehyde-3-phosphate dehydrogenase |
| HPRT1 | Hypoxanthine phosphoribosyltransferase 1 |
| RPLP0 | Ribosomal protein, large, P0 |
