# Supplementary material for: Pre-Exposure to Defibrotide Prevents Endothelial Cell Activation by Lipopolysaccharide: An Ingenuity Pathway Analysis
Source: Front Immunol. 2020 Dec 3;11:585519. doi: 10.3389/fimmu.2020.585519 (PMC7744778; doi:10.3389/fimmu.2020.585519)
Supplement: Supplementary file 2 [file Table_2.docx]

**Supplementary Table 2.** Genes regulation comparing to control group.

|  | **DFB** |  | **LPS** | | **LPS+DFB** | | **preDFB- LPS+DFB** | |
| --- | --- | --- | --- | --- | --- | --- | --- | --- |
| **Symbol** | Fold regulation | *p*-value | Fold regulation | *p*-value | Fold regulation | *p*-value | Fold regulation | *p*-value |
| **ACE** | 1.11 | 0.887 | -1.75 | 0.943 | -1.14 | 0.906 | -1.84 | 0.924 |
| **ADAM17** | -1.08 | 0.622 | 1.37 | 0.193 | 1.31 | 0.111 | 1.22 | 0.147 |
| **AGT*** | 2.02 | 0.212 | 1.07 | 0.838 | 1.27 | 0.693 | -2.86 | **0.071** |
| **AGTR1*** | 2.02 | 0.212 | 1.07 | 0.838 | 1.27 | 0.693 | -2.86 | **0.071** |
| **ALOX5** | 1.40 | 0.281 | -1.08 | 0.744 | 1.25 | 0.607 | -1.24 | 0.264 |
| **ANGPT1** | 1.32 | 0.731 | -1.28 | 0.463 | 1.04 | 0.775 | -3.49 | **0.089** |
| **ANXA5** | 1.31 | **0.040** | 1.38 | **0.043** | 1.30 | **0.006** | 1.36 | **0.020** |
| **APOE** | 1.18 | 0.502 | -1.29 | 0.489 | 1.09 | 0.646 | -1.36 | 0.395 |
| **BAX** | 1.56 | **0.024** | -1.07 | 0.837 | 1.34 | 0.101 | -1.40 | **0.076** |
| **BCL2** | 2.15 | 0.307 | 1.14 | 0.588 | 1.70 | 0.365 | -1.82 | 0.721 |
| **BCL2L1** | 1.54 | 0.127 | -1.17 | 0.986 | 1.26 | 0.339 | -1.38 | 0.526 |
| **CALCA** | 2.15 | 0.204 | 1.18 | 0.866 | 1.33 | 0.672 | -2.75 | **0.073** |
| **CASP1** | -1.37 | 0.560 | 2.18 | 0.267 | 1.60 | 0.340 | 3.66 | **0.005** |
| **CASP3** | 1.23 | 0.509 | 1.45 | 0.101 | 1.41 | 0.206 | 1.08 | 0.928 |
| **CAV1** | 1.02 | 0.691 | 1.55 | **0.076** | 1.47 | **0.075** | 1.39 | 0.092 |
| **CCL2** | -1.25 | 0.844 | 17.58 | **0.001** | 15.59 | **0.001** | 12.55 | **0.012** |
| **CCL5** | 1.60 | 0.362 | 42.85 | 0.146 | 59.54 | 0.119 | 4.60 | 0.112 |
| **CDH5** | 1.69 | 0.246 | -1.11 | 0.649 | 1.40 | 0.299 | -2.61 | 0.168 |
| **CFLAR** | 1.40 | **0.097** | 1.85 | **0.029** | 2.37 | **0.003** | 1.07 | 0.708 |
| **COL18A1** | 1.44 | 0.608 | -1.14 | 0.830 | 1.43 | 0.581 | -3.10 | **0.081** |
| **CX3CL1** | 2.40 | **0.060** | 20.07 | **0.022** | 37.84 | 0.009 | 2.95 | 0.291 |
| **EDN1** | 1.26 | 0.275 | 1.33 | 0.172 | 1.52 | **0.035** | 1.21 | 0.309 |
| **EDN2*** | 2.02 | 0.212 | 1.07 | 0.838 | 1.27 | 0.693 | -2.86 | **0.071** |
| **EDNRA** | 2.06 | 0.422 | 1.00 | 0.499 | 2.91 | 0.301 | -3.40 | **0.073** |
| **ENG** | 1.15 | 0.675 | -1.61 | 0.587 | -1.15 | 0.645 | -1.93 | 0.104 |
| **F2R** | 1.06 | 0.774 | -1.43 | 0.222 | -1.08 | 0.615 | -1.60 | **0.050** |
| **F3** | 1.03 | 0.730 | 9.63 | **0.038** | 8.25 | **0.020** | 6.75 | **0.010** |
| **FAS** | 1.43 | 0.276 | 1.34 | 0.266 | 1.26 | 0.405 | -1.41 | 0.231 |
| **FASLG** | 1.95 | 0.206 | 1.24 | 0.974 | 1.41 | 0.508 | -2.82 | **0.062** |
| **FGF1** | 1.26 | 0.601 | 1.18 | 0.775 | 1.34 | 0.474 | -2.03 | 0.260 |
| **FGF2** | 1.51 | 0.249 | 1.58 | 0.303 | 1.37 | 0.441 | -1.11 | 0.558 |
| **FLT1** | 1.77 | **0.071** | 1.04 | 0.866 | 1.40 | 0.253 | -1.73 | **0.040** |
| **FN1** | 1.54 | 0.273 | -1.07 | 0.920 | 1.34 | 0.538 | -2.62 | **0.052** |
| **HIF1A** | 1.62 | 0.109 | 1.61 | 0.089 | 1.91 | 0.045 | 1.89 | **0.031** |
| **HMOX1** | 2.11 | 0.119 | 1.65 | 0.263 | 2.27 | 0.129 | -1.13 | 0.679 |
| **ICAM1** | 1.24 | 0.505 | 18.65 | **0.001** | 21.81 | **0.001** | 8.08 | **0.004** |
| **IL11** | 1.58 | 0.959 | -1.04 | 0.519 | 1.47 | 0.698 | -3.37 | 0.211 |
| **IL1B** | 1.53 | 0.184 | 2.57 | **0.047** | 4.12 | **0.010** | 2.85 | 0.207 |
| **IL3** | 2.45 | **0.075** | 1.18 | 0.975 | 1.54 | 0.272 | -2.43 | 0.279 |
| **IL6** | -1.47 | 0.414 | 12.75 | **0.002** | 14.73 | **0.006** | 6.35 | **0.008** |
| **IL7** | 1.57 | 0.277 | 38.66 | **0.013** | 61.80 | **0.005** | 6.61 | **0.055** |
| **ITGA5** | 1.38 | 0.325 | -1.18 | 0.897 | 1.12 | 0.629 | -2.04 | **0.025** |
| **ITGAV** | 1.35 | 0.211 | 1.46 | **0.099** | 1.99 | **0.010** | 1.07 | 0.964 |
| **ITGB1** | 1.31 | 0.251 | 1.09 | 0.778 | 1.15 | 0.578 | 1.15 | 0.583 |
| **ITGB3** | 1.24 | 0.323 | 1.28 | 0.408 | 1.50 | 0.169 | 1.06 | 0.799 |
| **KDR** | 1.73 | 0.553 | 1.09 | 0.857 | 1.34 | 0.747 | 1.34 | 0.750 |
| **KIT** | 1.51 | 0.786 | 2.65 | **0.094** | 2.87 | **0.076** | 5.80 | 0.113 |
| **KLK3*** | 2.02 | 0.212 | 1.31 | 0.931 | 1.47 | 0.600 | -2.69 | **0.074** |
| **MMP1** | -14.19 | 0.281 | 1.73 | 0.390 | 1.18 | 0.817 | 4.85 | 0.913 |
| **MMP2** | 1.07 | 0.957 | -1.33 | 0.345 | -1.11 | 0.702 | -2.21 | **0.041** |
| **MMP9** | 1.64 | 0.232 | 1.01 | 0.936 | 1.30 | 0.546 | -2.36 | **0.091** |
| **NOS3** | 1.17 | 0.576 | -2.07 | 0.064 | -1.84 | 0.101 | -2.56 | **0.020** |
| **NPPB** | 2.22 | 0.111 | 1.01 | 0.764 | 1.53 | 0.227 | -2.70 | 0.108 |
| **NPR1** | 1.23 | 0.746 | -1.30 | 0.987 | 1.40 | 0.656 | -2.08 | 0.599 |
| **OCLN** | 1.13 | 0.749 | -1.36 | 0.353 | -1.61 | 0.087 | 1.43 | 0.107 |
| **PDGFRA** | 2.12 | 0.132 | 2.66 | 0.052 | 3.47 | **0.044** | -1.11 | 0.587 |
| **PECAM1** | -1.21 | 0.739 | 1.17 | 0.530 | -1.22 | 0.917 | 3.29 | 0.144 |
| **PF4** | 1.85 | 0.301 | 2.39 | 0.258 | 3.09 | 0.208 | 1.38 | 0.421 |
| **PGF** | 2.72 | 0.115 | 1.21 | 0.788 | 1.34 | 0.499 | -3.32 | 0.134 |
| **PLAT** | 1.65 | 0.245 | 1.10 | 0.594 | 1.55 | 0.381 | -1.16 | 0.448 |
| **PLAU** | 1.07 | 0.975 | 1.66 | 0.209 | 2.24 | **0.095** | -1.30 | 0.716 |
| **PLG*** | 2.02 | 0.213 | 1.11 | 0.847 | 1.27 | 0.693 | -2.86 | **0.071** |
| **PROCR** | 1.08 | 0.823 | -1.55 | 0.247 | -1.12 | 0.698 | -2.37 | **0.017** |
| **PTGIS** | -1.38 | 0.820 | -1.16 | 0.724 | 1.13 | 0.916 | -1.47 | 0.187 |
| **PTGS2** | 1.01 | 0.907 | 1.89 | 0.199 | 3.62 | **0.027** | -1.20 | 0.411 |
| **PTK2** | 1.26 | 0.227 | -1.02 | 0.987 | 1.22 | 0.294 | -1.15 | 0.439 |
| **SELE** | 1.16 | 0.632 | 43.24 | 0.021 | 81.93 | 0.006 | 7.90 | **0.019** |
| **SELL** | 1.01 | 0.758 | -1.26 | 0.426 | 1.43 | 0.518 | 1.42 | 0.613 |
| **SELPLG** | -1.19 | 0.639 | -2.02 | **0.049** | -1.33 | 0.267 | 1.57 | 0.147 |
| **SERPINE1** | 1.83 | 0.132 | 1.56 | 0.187 | 2.09 | 0.064 | -1.27 | 0.681 |
| **SOD1** | 1.44 | 0.211 | 1.47 | 0.197 | 1.27 | 0.422 | 1.66 | **0.081** |
| **SPHK1** | 2.71 | **0.079** | 1.05 | 0.253 | 2.07 | 0.141 | -3.98 | **0.044** |
| **TEK** | 1.01 | 0.894 | -1.30 | 0.510 | -1.12 | 0.804 | -1.26 | 0.379 |
| **TFPI** | -1.28 | 0.507 | -1.09 | 0.988 | -1.15 | 0.847 | 1.41 | 0.357 |
| **TGFB1** | 1.09 | 0.882 | -1.60 | 0.227 | -1.17 | 0.455 | -2.12 | **0.028** |
| **THBD** | 1.27 | 0.830 | -2.97 | 0.426 | -2.22 | 0.339 | -4.66 | 0.220 |
| **THBS1** | 1.04 | 0.783 | -1.25 | 0.461 | 1.04 | 0.935 | -1.39 | **0.091** |
| **TIMP1** | 1.19 | 0.346 | -1.31 | 0.511 | 1.13 | 0.457 | -1.33 | 0.138 |
| **TNF*** | 2.02 | 0.213 | 2.58 | 0.158 | 3.73 | 0.108 | 1.30 | 0.817 |
| **TNFSF10** | 1.06 | 0.871 | 2.33 | 0.092 | 2.37 | **0.071** | 2.95 | **0.029** |
| **TYMP** | 1.55 | 0.635 | 1.47 | 0.348 | 1.90 | 0.277 | -2.16 | 0.124 |
| **VCAM1** | 1.02 | 0.545 | 39.28 | **0.004** | 39.41 | **0.002** | 23.78 | **0.019** |
| **VEGFA** | 1.44 | 0.444 | 2.43 | **0.057** | 2.33 | **0.078** | 2.49 | **0.031** |
| **VWF** | 1.35 | 0.490 | -1.13 | 0.712 | 1.09 | 0.946 | -1.03 | 0.676 |

*This gene's average threshold cycle is either not determined or greater than the defined cut-off (default 35) in the investigated sample and control, meaning that its expression was undetected, making this fold-change result erroneous and un-interpretable. P values ≤0.1 are in bold.
